# The adenosine A_2A_ receptor antagonist KW6002 distinctly regulates retinal ganglion cell morphology during postnatal development and neonatal inflammation

**DOI:** 10.3389/fphar.2022.1082997

**Published:** 2022-12-16

**Authors:** Shisi Hu, Yaoyao Li, Yuanjie Zhang, Ruyi Shi, Ping Tang, Di Zhang, Xiuli Kuang, Jiangfan Chen, Jia Qu, Ying Gao

**Affiliations:** ^1^ The Molecular Neuropharmacology Laboratory and the Eye-Brain Research Center, State Key Laboratory of Ophthalmology, Optometry and Visual Science, Wenzhou Medical University, Wenzhou, China; ^2^ State Key Laboratory of Ophthalmology, Optometry and Visual Science, Wenzhou Medical University, Wenzhou, China; ^3^ School of Ophthalmology and Optometry and Eye Hospital, Wenzhou Medical University, Wenzhou, China; ^4^ Hainan Eye Hospital and Key Laboratory of Ophthalmology, Zhongshan Ophthalmic Center, Sun Yat-sen University, Haikou, China

**Keywords:** adenosine A_2A_ receptor, retinal ganglion cell, morphology, 3D reconstruction, development, neonatal inflammation

## Abstract

Adenosine A_2A_ receptors (A_2A_Rs) appear early in the retina during postnatal development, but the roles of the A_2A_Rs in the morphogenesis of distinct types of retinal ganglion cells (RGCs) during postnatal development and neonatal inflammatory response remain undetermined. As the RGCs are rather heterogeneous in morphology and functions in the retina, here we resorted to the Thy1-YFPH transgenic mice and three-dimensional (3D) neuron reconstruction to investigate how A_2A_Rs regulate the morphogenesis of three morphologically distinct types of RGCs (namely Type I, II, III) during postnatal development and neonatal inflammation. We found that the A_2A_R antagonist KW6002 did not change the proportion of the three RGC types during retinal development, but exerted a bidirectional effect on dendritic complexity of Type I and III RGCs and cell type-specifically altered their morphologies with decreased dendrite density of Type I, decreased the dendritic field area of Type II and III, increased dendrite density of Type III RGCs. Moreover, under neonatal inflammation condition, KW6002 specifically increased the proportion of Type I RGCs with enhanced the dendrite surface area and volume and the proportion of Type II RGCs with enlarged the soma area and perimeter. Thus, A_2A_Rs exert distinct control of RGC morphologies to cell type-specifically fine-tune the RGC dendrites during normal development but to mainly suppress RGC soma and dendrite volume under neonatal inflammation.

## 1 Introduction

Adenosine, an endogenous nucleoside, is a neuromodulator and intracellular messenger, which is widely present in the central nervous system, including the retina. Adenosine can modulate neuronal excitability, neurotransmitter release and synaptic activity by acting at four subtypes of adenosine receptors, namely A1, A_2A_, A_2B_, and A3 receptors (Chen et al., 2013). Among them, adenosine A_2A_ receptor (A_2A_R) appears early in the retina during development, which is detected at embryonic day 6 in chick embryo retina (Brito et al., 2012) and is also expressed widely in the retina, such as photoreceptors, inner nuclear layer neurons, starburst amacrine cells and retinal ganglion cells (RGCs). Previous studies have shown that the retinal A_2A_Rs exert control of dark-adaption in regulating photoreceptor coupling (Li et al., 2013), expression of rod opsin mRNA in tiger salamander (Alfinito et al., 2002), the release of glutamate from rod photoreceptors (Stella et al., 2003), the generation of the electroretinogram a- and b-waves and oscillatory potentials (OPs) (Jonsson and Eysteinsson, 2017) and the generation and modulation of retinal waves (Huang et al., 2014).

Both *in vitro* and *in vivo* studies have revealed that A_2A_Rs play important roles in brain development (Silva et al., 2013; Ribeiro et al., 2016; Alcada-Morais et al., 2021). Our previous study has found that A_2A_Rs modulate microglia-mediated synaptic pruning of the retinogeniculate pathway in the dorsal lateral geniculate during postnatal development (Miao et al., 2021). However, the exact role of the A_2A_Rs on the development of retinal neurons including different RGC types is still not known. The RGCs are rather heterogeneous and have been classified into over 30 different types, based on their dendritic anatomies, functional characteristics or transcriptomic features (Baden et al., 2016; Bae et al., 2018; Goetz et al., 2022; Huang et al., 2022). Furthermore, retinal A_2A_Rs also participate in not only the normal retinal development but also the development under pathological processes in the retina, such as neuroinflammation and inflammation-associated retinal degeneration. While the involvement of adenosine and A_2A_R in the regulation of brain microglia in two neonatal rat models of neuroinflammation (Colella et al., 2018) has been studied, much less attention has been paid to the effects of A_2A_Rs on the development of RGCs after neonatal inflammation.

In the present study, we investigated how A_2A_Rs regulate the morphology of RGCs during retinal development and neonatal inflammation, using the Thy1-YFPH transgenic mice coupled with three-dimensional (3D) neuron reconstruction method. We demonstrated that during normal development, the A_2A_R antagonist KW6002 mainly decreased RGC morphogenesis as evident by the reduced dendritic field area of Type II and III RGCs, and the reduced dendrite density of Type I but with the increased the dendrite density of Type III RGCs. Moreover, under neonatal inflammation, KW6002 specifically increased the proportion of Type I RGCs with enhanced the dendrite surface area and volume and the proportion of Type II RGCs with enlarged the soma area and perimeter. Thus, A_2A_Rs distinctly regulate RGC morphologies by fine-tuning the RGC dendrites in a cell type-specific manner during normal development, but mainly suppressing RGC soma and dendrite volume under neonatal inflammation.

## 2 Materials and methods

### 2.1 Animals

All animal protocols were approved by the Animal Care Committee of Wenzhou Medical University. The YFPH line of transgenic mice was obtained from the Jackson Laboratory (strain: B6. Cg-Tg (Thy1-YFPH) 2Jrs/J; Bar Harbor, Maine). All mice were given *ad libitum* access to food and water under a 12 h light/dark cycle with 50–60% humidity. The day of birth was counted as postnatal day 0 (P0).

The littermates of the Thy1-YFPH mice were randomly divided into two groups. Pups received intraperitoneal (IP) injections of the A_2A_R antagonist KW6002 (10 mg/kg body weight, freshly prepared in dimethyl sulfoxide (DMSO, Sigma), ethoxylated castor oil (Sigma), and phosphate-buffered saline (PBS) with a proportion of 15%:15%:70% (Miao et al., 2021)) every day from P4 to P6. The control group was administered the corresponding vehicle in the same volume. The neonatal inflammation was induced in Thy1-YFPH mice by an intraperitoneal injection of lipopolysaccharide (LPS, 1 mg/kg, *E. coli* 055: B5; Sigma) 4 min after KW6002 treatment at P4.

### 2.2 Immunohistochemistry of retinal whole-mounts

Immunohistochemistry experiments were carried out as previously described (Gao et al., 2018). Briefly, after the Thy1-YFPH mice were anesthetized, the eyes were enucleated on P21 and fixed in 4% paraformaldehyde (PFA) for 30 min. The retinas were isolated from eyeballs, fixed in 4% PFA for additional 10 min, and incubated with 3% H_2_O_2_ for 20 min. After being blocked in a blocking solution (5% normal donkey serum plus 1% BSA, 0.2% glycine, 0.2% lysine, and 0.3% Triton X-100) for 2 h at room temperature, retinas were incubated with goat polyclonal antibodies against GFP (1:500, NB100-1770, Novus Biologicals) for 2 days at 4°C. Then the retinas were sequentially incubated with the biotinylated donkey anti-goat antibodies, the avidin-biotin complex (Vectastain ABC Elite Kit; Vector Laboratories, USA), the 3,3′-diaminobenzidine (DAB tablets, Sigma), and finally flat-mounted on glass slides with aqueous mounting medium (IMMCO Diagnostics, Inc).

### 2.3 3D reconstruction and quantitative morphometry

The fully and strongly stained YFP-positive RGCs (that achieve the standard for the detailed morphological analyses) were randomly selected and reconstructed, while those with faint staining and uncompleted structure were discarded. The morphology of RGCs was reconstructed by using Neurolucida system (MicroBrightField Inc., USA) and a bright-field light microscope (Zeiss, Germany) at a magnification of ×63, as previously described (Gao et al., 2018). A battery of morphological parameters were extracted from 251 fully reconstructed RGCs by the NeuroExplorer (MicroBrightField Inc., USA). Sholl analysis on dendrites of RGCs was also performed using “Sholl Analysis” in Neuroexplorer (Sholl, 1953). The spatial distributions of dendritic intersection with the concentric circles were quantified in terms of stepped distance circle regions (10 μm) from the soma.

### 2.4 Statistical analysis

Results were expressed as mean ± standard error of mean (SEM). Kruskal-Wallis one-way ANOVA (k samples), independent Student’s *t*-test and chi-square test were performed by SPSS 26. The significant level was set at *p* < 0.05 for all comparisons.

## 3 Results

### 3.1 The A_2A_R antagonist KW6002 did not alter the proportion of RGC morphological types during the development

**FIGURE 1:**
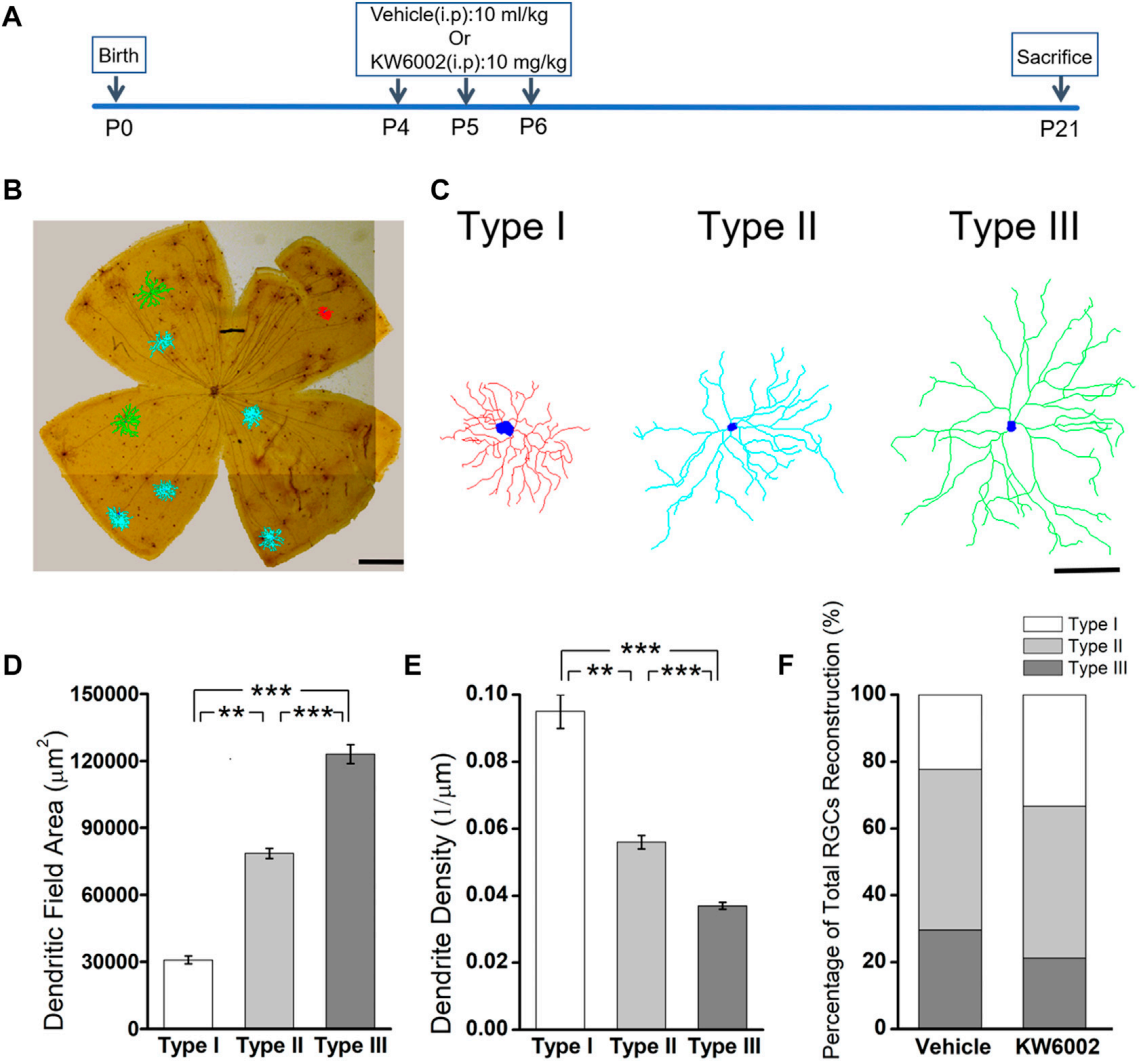
The effect of A_2A_R antagonist KW6002 on the proportion of different morphological types of RGCs during the development. **(A)** Timeline of KW6002 (or vehicle) administration in transgenic Thy-1 YFPH mice. **(B)** Representative flat-mounted whole retina showing three different morphological types of RGCs in different colors from transgenic Thy-1 YFPH mice (red, Type I; blue, Type II; green, Type III). The morphology of an individual RGC was revealed by immunohistochemistry and reconstructed by the Neurolucida system. Scale bar, 500 μm. **(C)** Representative 3D reconstructions of the three morphological types of RGCs as shown in B. Scale bar, 100 μm.**(D,E)** Quantitative evaluation of dendritic field area **(D)** and dendrite density **(E)** among different morphological types of RGCs. **(F)** The proportion of different morphological types of RGCs from the control and KW6002-treated mice during the development. Values are mean ± SEM; **p < 0.01, ***p < 0.001. 54 RGCs from six retinas (3 vehicle-treated mice) and 66 RGCs from eight retinas (4 KW6002-treated mice) were analyzed in the control and KW6002-treated group, respectively. On average, nine RGCs were analyzed in each eye in the control group, while 8.25 RGCs per eye were analyzed in KW6002-treated group.

Since the RGCs are rather heterogeneous in the retina, we take advantage of the Thy-1 YFPH transgenic line, which expresses the yellow fluorescent protein (YFP) only in a fraction of RGCs (Barnstable and Dräger, 1984; Feng et al., 2000), to study the effect of A_2A_R on the development of RGCs. During retinal development, Thy-1 YFPH neonates received intraperitoneal injections of the A_2A_R antagonist KW6002 from P4 to P6 and were sacrificed at P21 **(** Figure 1A). 3D reconstruction of well-stained YFP^+^ cells (n = 120 cells) for detailed morphometric analyses was performed in the retina at P21 **(** Figure 1B). As described previously, we classified these Thy1-positive RGCs labeled with YFP into three major morphological classes (Type I, II and III), based on the morphological features of the dendritic field and dendrite density (Gao et al., 2018). Type I had a small dendritic field area and high dendrite density, whereas Type III had a large dendritic field area but low dendrite density. Type II was just between Type I and III **(** Figure 1C). The quantitative analysis further confirmed the significant differences among the three RGC types (**p < 0.01, ***p < 0.001, Figures 1D,E). Compositions of the three RGC types were similar between the KW6002-treated and control groups (Type I: control, n = 12, 22.22% vs. KW6002, n = 22, 33.33%; Type II: control, n = 26, 48.15% vs. KW6002, n = 30, 45.45%; Type III: control, n = 16, 29.63% vs. KW6002, n = 14, 21.21%; *p* > 0.05; Figure 1F). Therefore, KW6002 had no effect on the proportion of these RGC morphological types during retinal development.

### 3.2 KW6002 mainly decreased RGC morphogenesis by the reduced dendritic field area of Type II and III RGCs, and the reduced dendrite density of Type I but with the increased dendrite density of Type III RGCs

**FIGURE 2:**
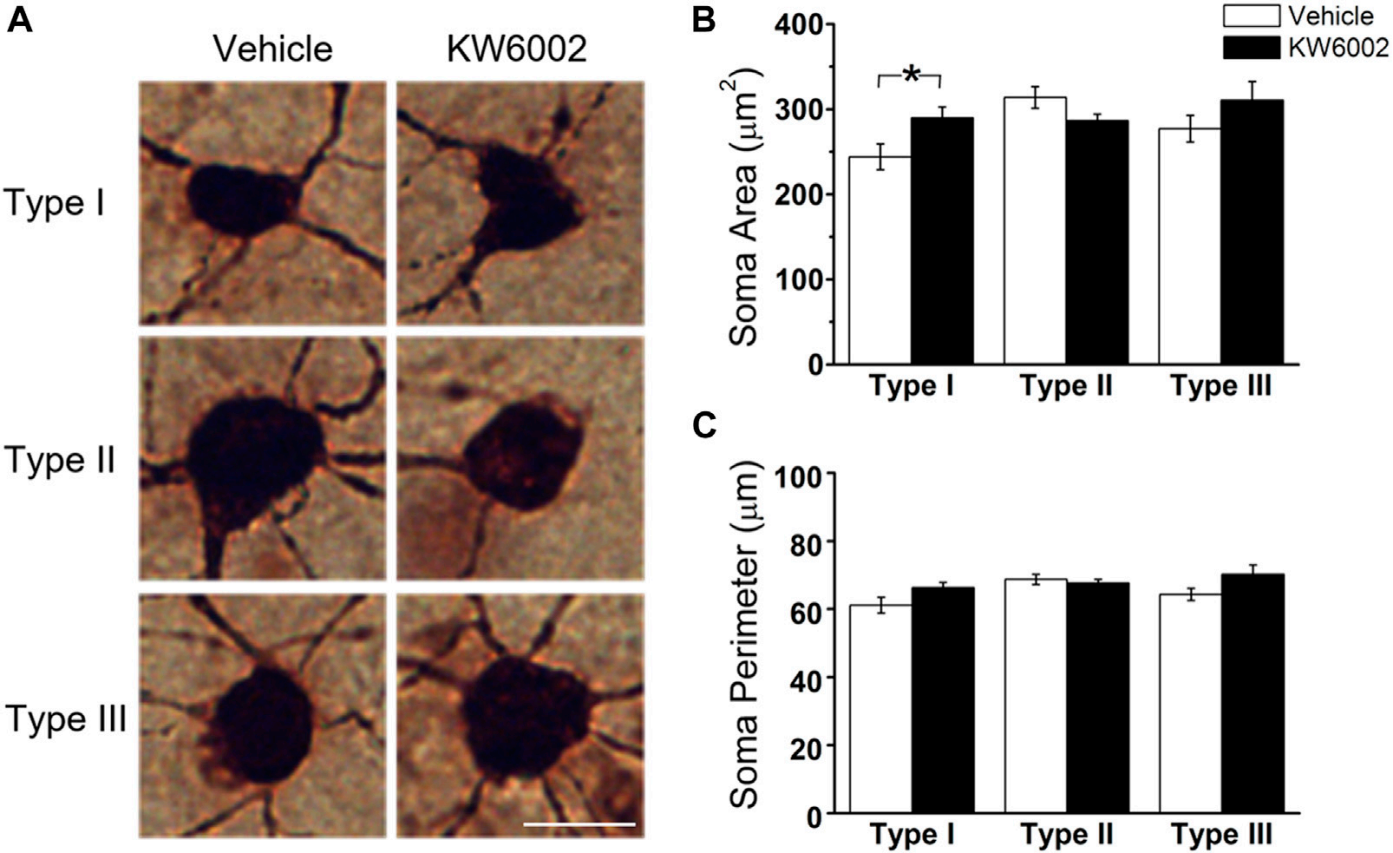
The effect of KW6002 on the somatic morphology of the three RGC types during the development. **(A)** Representative pictures of somata in each RGC type examined from vehicle -treated and KW6002-treated group. Scale bar, 20 μm. **(B**,**C)** Comparative analysis of soma area **(B)** and perimeter **(C)** of different RGC types between vehicle-treated and KW6002-treated mice. Data represent mean ± SEM. *p < 0.05.

The RGC somata have different shapes, such as triangular, round, and oval. RGCs with different morphological shapes are used to the different parameters. We firstly studied the somatic development of RGCs and found that KW6002 significantly increased the soma area by 18.69% in Type I (control, 244.04 ± 15.10 μm^2^ vs. KW6002, 289.66 ± 13.04 μm^2^; *p < 0.05; Figures 2A,B and Supplementary Table S1), while no significant effect was found on Type II and III (*p* > 0.05; Figures 2A,B). Meanwhile, no significant difference was found in the soma perimeter of the three morphological types of RGCs by KW6002 (*p* > 0.05; Figures 2A,C). These results indicate that A_2A_Rs can differentially modulate the somatic development of RGCs during retinal development.

**FIGURE 3:**
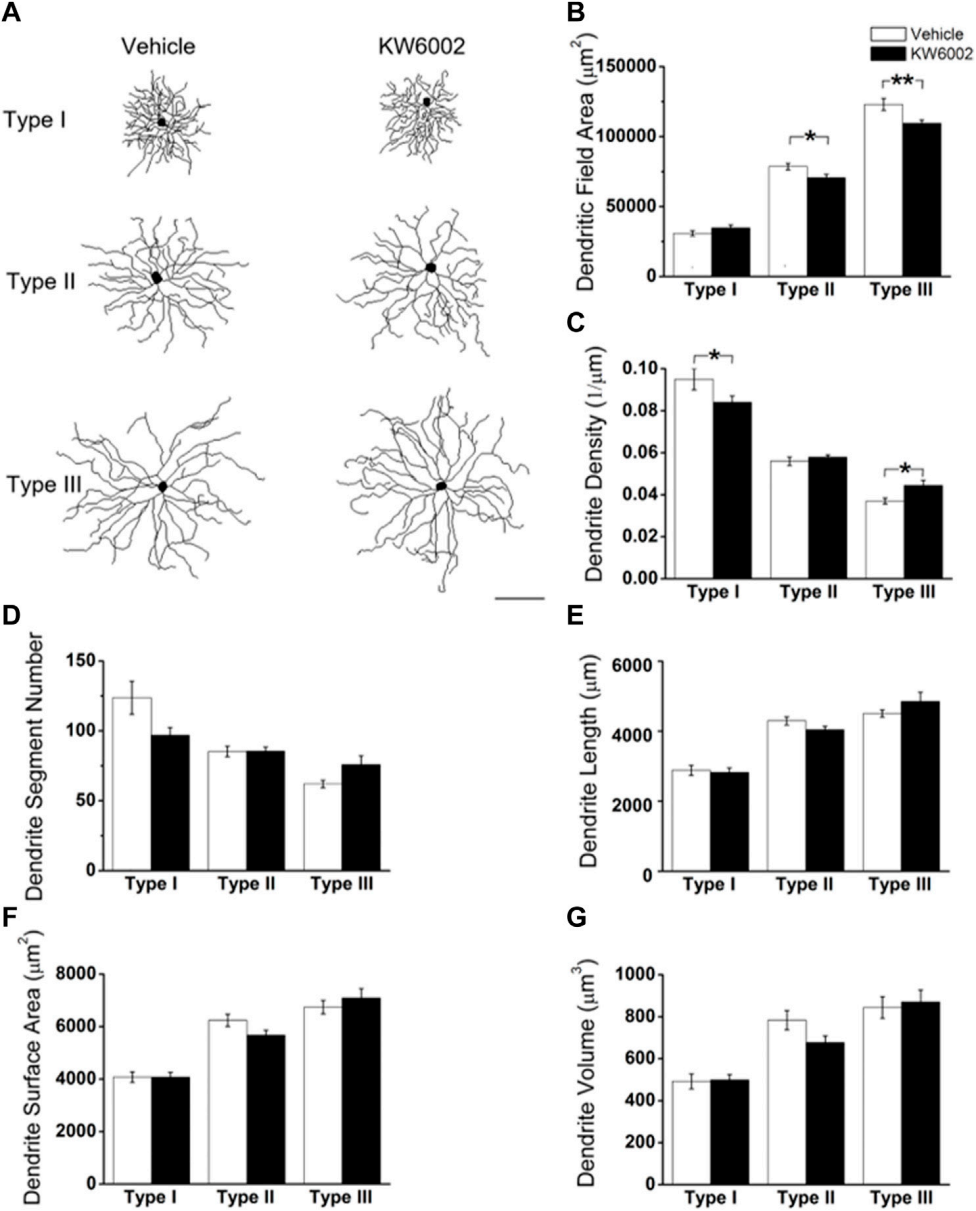
KW6002 differentially affected the dendritic morphology of RGCs during the development. **(A)** Representative 3D reconstructions of the three RGC types from the vehicle-treated and KW6002-treated mice. **(B–G)** Comparative analysis of dendritic field area **(B)**, dendrite density **(C)**, dendrite segment number **(D)**, dendrite length **(E)**, dendrite surface area **(F)** and dendrite volume **(G)** in each RGC type between the vehicle-treated and KW6002-treated group. Data represent mean ± SEM. *p < 0.05; **p < 0.01. Scale bar, 100 μm.

We further compared the morphological features of dendrites, such as dendritic field area, dendrite density, segment number, length, surface area and volume etc, between the two groups (Figure 3). The dendritic field area was diminished by 10.13% and 10.99%, respectively, in Type II (control, 78,610.04 ± 2295.94 μm^2^ vs. KW6002, 70,646.83 ± 2472.02 μm^2^; *p < 0.05; Figures 3A,B) and Type III RGCs (control, 123,035.42 ± 4269.09 μm^2^ vs. KW6002, 109,513.94 ± 2438.7 μm^2^; **p < 0.01; Figures 3A,B) after KW6002 treatment, whereas no significant effect was found in Type I (*p >* 0.05; Figure 3B). Compared to the control group, KW6002 attenuated the dendrite density by 12.00% (control, 9.50 ± 0.48 (×10^–2^, 1/µm) vs. KW6002, 8.36 ± 0.28 (×10^–2^, 1/µm); *p < 0.05; Figure 3C) in Type I RGCs, but induced 19.62% enhancement of the dendrite density (control, 3.72 ± 0.14 (×10^–2^, 1/µm) vs. KW6002, 4.45 ± 0.24 (×10^–2^, 1/µm); *p < 0.05; Figure 3C) in Type III RGCs. KW6002 didn’t change the dendrite segment number and total dendrite length of all three RGC types during normal development (*p >* 0.05; Figures 3D,E). As to the dendrite surface area and volume, no significant effect was found in each RGC type after KW6002 treatment during normal development (*p >* 0.05; Figures 3F,G). These results implied that A_2A_Rs can reorganize the dendritic architecture of RGCs in the retina, which is dependent on the RGC types.

**FIGURE 4:**
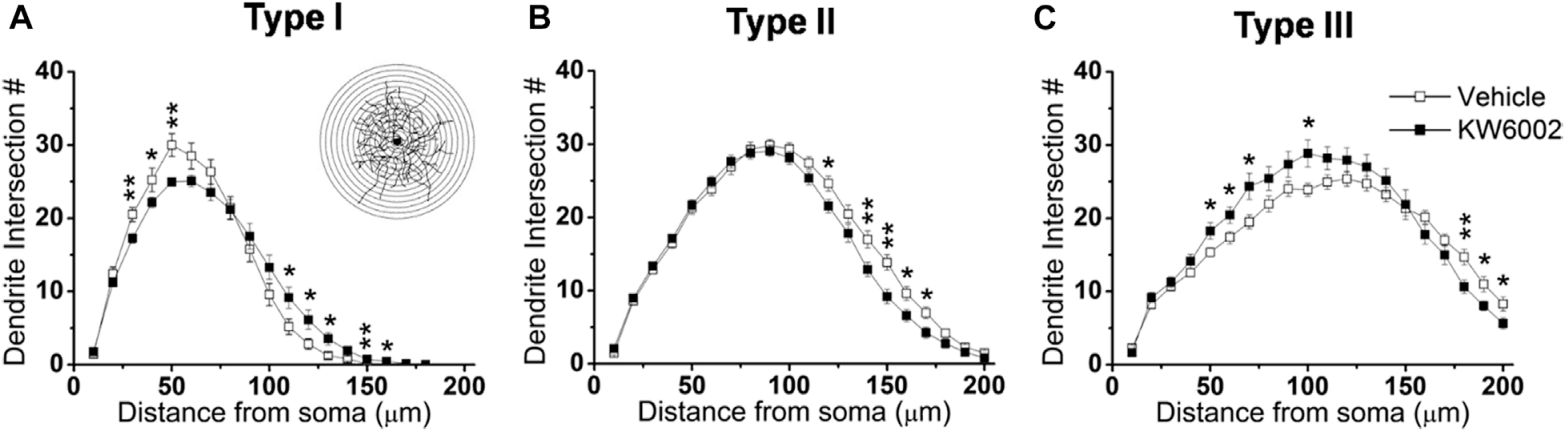
The dendritic complexity of RGCs was differentially altered by KW6002 during the development.**(A–C)** Quantitative Sholl analysis of dendrite intersection numbers in each RGC type from the vehicle-treated and KW6002-treated group during the development. Inset, a series of concentric circles with increasing radius at a 10 μm step were superimposed on the RGC. Data represent mean ± SEM. *p < 0.05; **p < 0.01.

To further investigate the effect of KW6002 on the spatial distribution of dendritic morphology, Sholl analyses were performed on quantifications of the distribution of dendritic intersections and revealed that KW6002 had a dual effect on Type I and III RGCs. KW6002 significantly decreased the dendritic intersection of Type I RGCs at 30–50 μm, but increased them at 110–160 μm from the soma (*p < 0.05 or **p < 0.01; Figure 4A) On the contrary, as to the Type III RGCs, the dendritic intersections were significantly increased at 50–70 μm and 100 μm but decreased at 180–200 μm from the soma (*p < 0.05 or **p < 0.01; Figure 4C) after KW6002 treatment. Meanwhile, KW6002 significantly decreased the dendritic intersection of Type II RGCs mainly at 120–170 μm, which is far from the soma (*p < 0.05 or **p < 0.01; Figure 4B). These results indicate the fine-tune effect of A_2A_Rs on the dendritic development of RGCs during the retinal development.

### 3.3 KW6002 changed the proportion of RGC morphological types after neonatal inflammation

**FIGURE 5:**
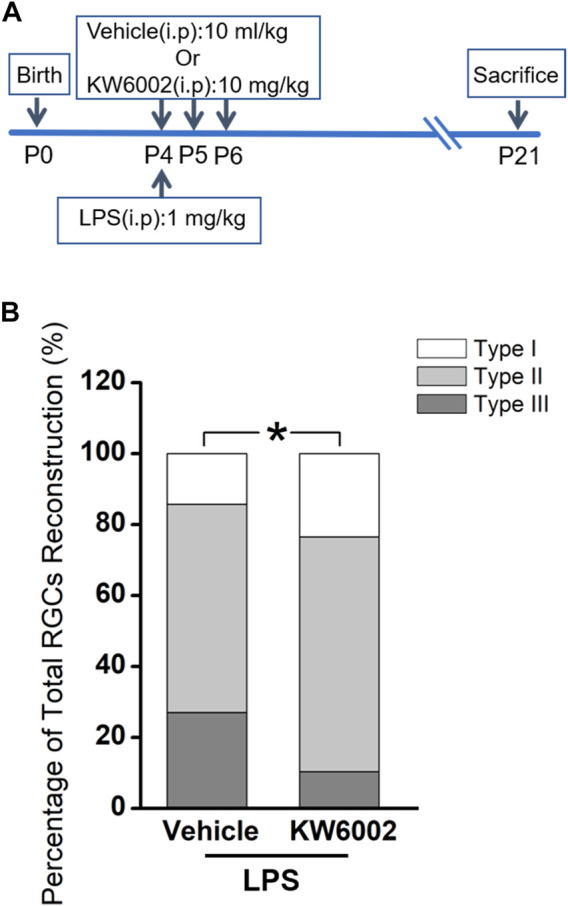
KW6002 changed the proportion of RGC morphological types after neonatal inflammation. **(A)** Timeline of KW6002 (or vehicle) administration with an intraperitoneal injection of LPS in transgenic Thy-1 YFPH mice. **(B)** The proportion of different morphological types of RGCs from the control and KW6002-treated mice after neonatal LPS exposure. *p < 0.05. 63 RGCs from 14 retinas (7 vehicle-treated mice) and 68 RGCs from 12 retinas (6 KW6002-treated mice) in the control and KW6002-treated group after neonatal LPS exposure, respectively. On average, 4.5 and 5.67 RGCs per eye were analyzed in each group.

Apart from its physiological role, we further studied the effects of A_2A_R on the development of RGCs after the neonatal inflammation. To induce the neonatal inflammation, neonates received a single intraperitoneal injection of LPS immediately after KW6002 treatment at P4. Then they were administrated with KW6002 in the same manner as that in the normal condition and were sacrificed at P21 (Figure 5A). The well-stained YFP^+^ cells (n = 131 cells) in the retina were 3D reconstructed for detailed morphometric analyses at P21. We found that after neonatal LPS exposure, the compositions of Type I and Type II RGCs significantly increased, but Type III decreased in the KW6002-treated groups, compared to the control groups (Type I: control, n = 9, 14.29% vs. KW6002, n = 16, 23.53%; Type II: control, n = 37, 58.73% vs. KW6002, n = 45, 66.18%; Type III: control, n = 17, 26.98% vs. KW6002, n = 7, 10.29%; *p < 0.05; Figure 5B). These results indicate that A_2A_Rs altered the compositions of the three RGC types after neonatal inflammation.

### 3.4 KW6002 increased the proportion of Type I RGCs with enhanced the dendrite surface area and volume and Type II RGCs with enlarged soma after neonatal inflammation

**FIGURE 6:**
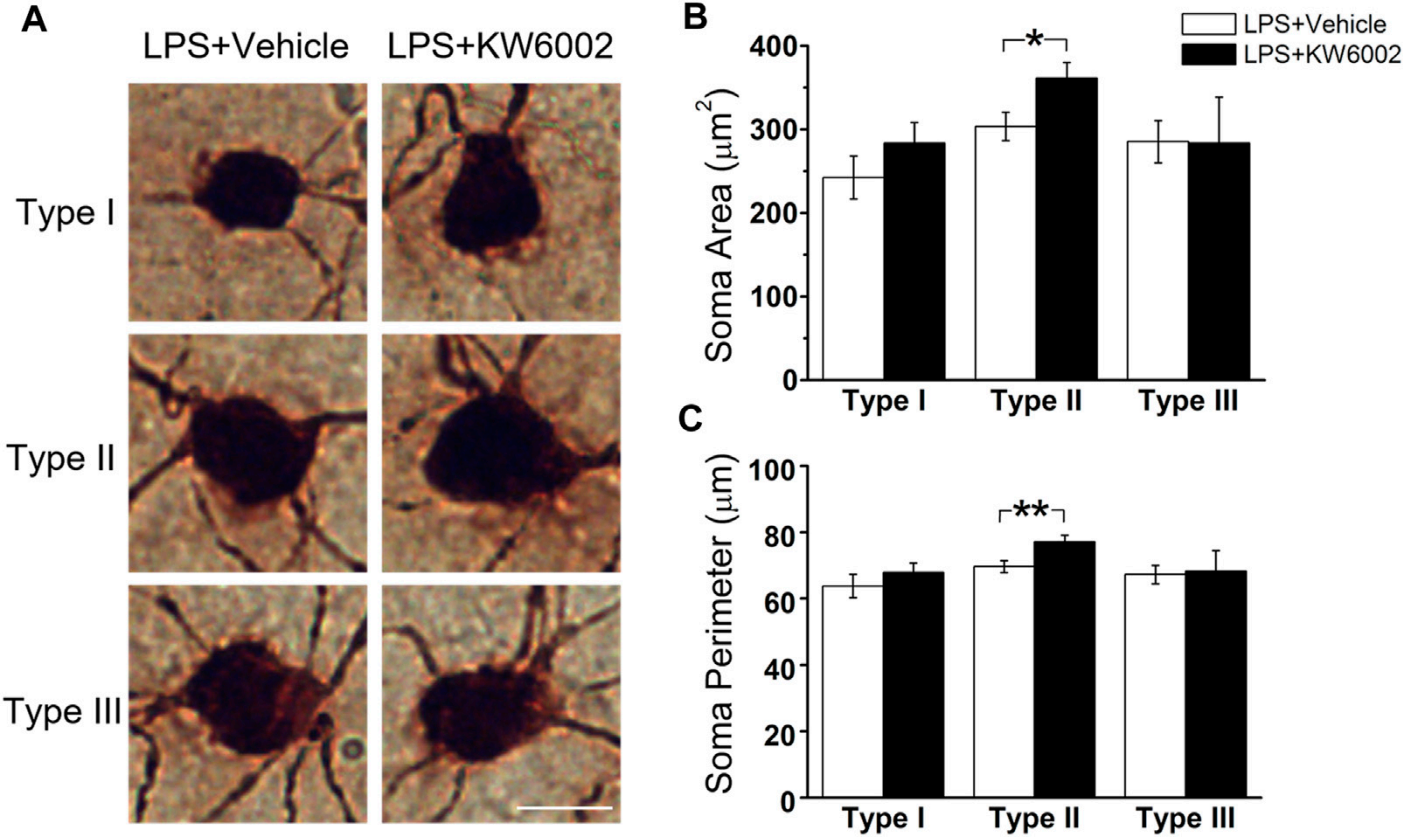
KW6002 enlarged the soma of Type II RGCs after neonatal LPS exposure. **(A)** Representative pictures of somata in each RGC type examined from vehicle -treated and KW6002-treated group after neonatal LPS exposure. Scale bar, 20 μm. **(B**,**C)** Comparative analysis of soma area **(B)** and perimeter **(C)** of different RGC types between vehicle-treated and KW6002-treated mice after neonatal LPS exposure. Data represent mean ± SEM. *p < 0.05; **p < 0.01.

**FIGURE 7:**
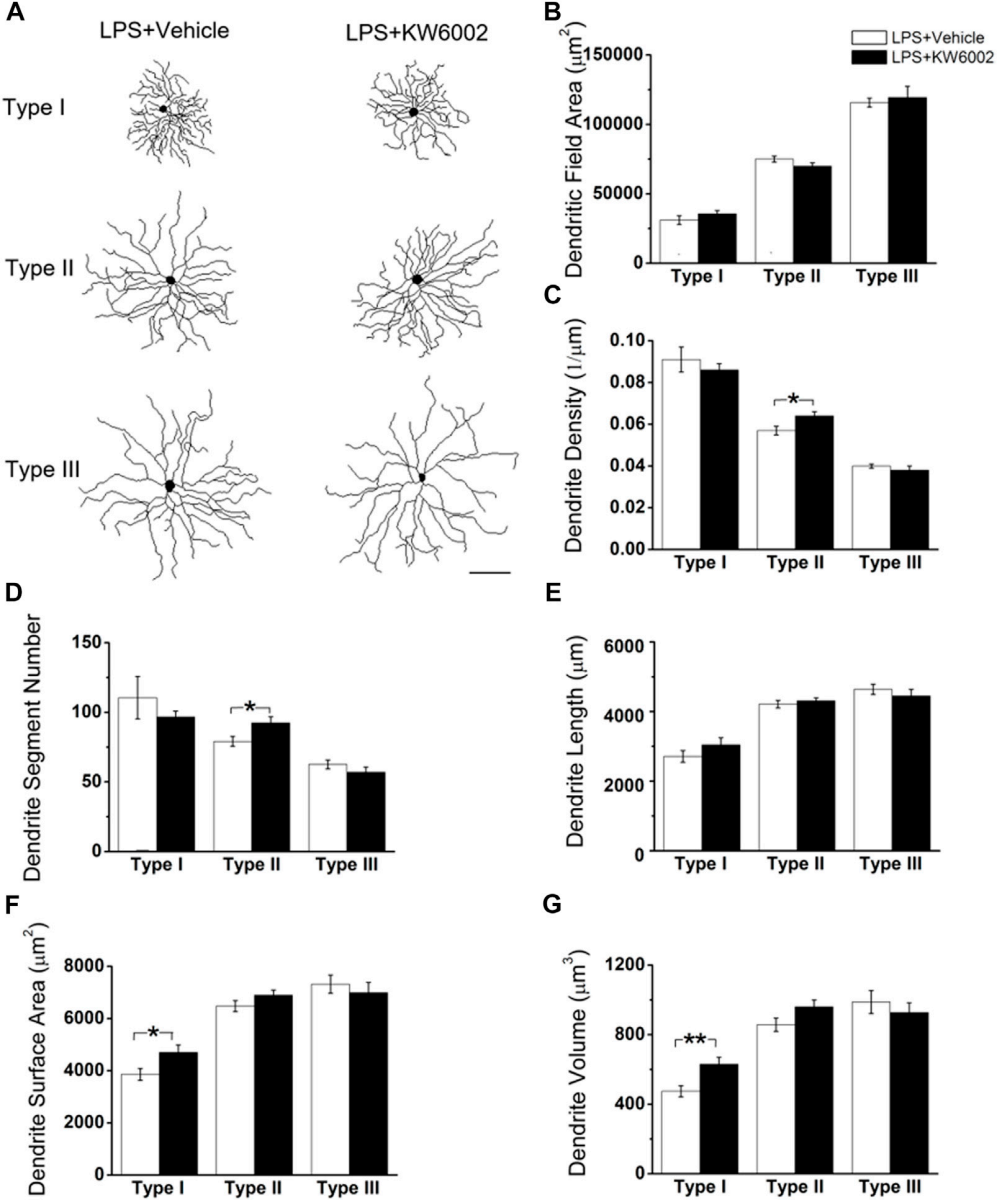
The effect of KW6002 on the dendritic morphology of RGCs after neonatal LPS exposure. **(A)** Representative 3D reconstructions of the three RGC types from the vehicle-treated and KW6002-treated mice after neonatal LPS exposure. **(B–G)** Comparative analysis of dendritic field area **(B)**, dendrite density **(C)**, dendrite segment number **(D)**, dendrite length **(E)**, dendrite surface area **(F)** and dendrite volume **(G)** in each RGC type between the vehicle-treated and KW6002-treated group after neonatal LPS exposure. Data represent mean ± SEM. *p < 0.05; **p < 0.01. Scale bar, 100 μm.

After LPS treatment, KW6002 significantly enlarged the soma area by 19.10% (control, 303.54 ± 16.96 μm^2^ vs. KW6002, 361.51 ± 18.69 μm^2^; *p < 0.05; Figures 6A,B and Supplementary Table S2) and the soma perimeter by 10.73% (control, 69.64 ± 1.79 μm vs. KW6002, 77.11 ± 2.01 μm; **p < 0.01; Figure 6C) in Type II. However, KW6002 had no significant effect on the soma area and perimeter of Type I and III after neonatal LPS exposure(*p >* 0.05; Figures 6B,C). Beside the soma, we also examined the dendritic morphology and found that the dendritic field area and total dendrite length of the three morphological types of RGCs were not affected by KW6002 after neonatal LPS exposure (*p >* 0.05; Figures 7A, B,E). Interestingly, KW6002 significantly augmented the dendrite density (control, 5.73 ± 0.17 (×10^–2^, 1/µm) vs. KW6002, 6.32 ± 0.19 (×10^–2^, 1/µm); *p < 0.05; Figure 7C) and segment number (control, 79.16 ± 3.50 vs. KW6002, 92.29 ± 4.03; *p < 0.05; Figure 7D) of Type II RGCs, but didn’t affect those of Type I and Type III RGCs after neonatal LPS exposure. Moreover, KW6002 significantly enhanced the dendrite surface area and volume of Type I RGCs (control, 3861.30 ± 227.05 μm^2^ vs. KW6002, 4700.80 ± 283.86 μm^2^; *p < 0.05; Figure 7F; control, 474.82 ± 31.88 μm^3^ vs. KW6002, 629.75 ± 40.26 μm^3^; **p < 0.01; Figure 7G) after neonatal exposure to LPS, while no significant change was found in the other two RGC types. These results suggest that A_2A_Rs induced differential alterations in the soma and dendritic architecture of RGCs after neonatal inflammation.

**FIGURE 8:**
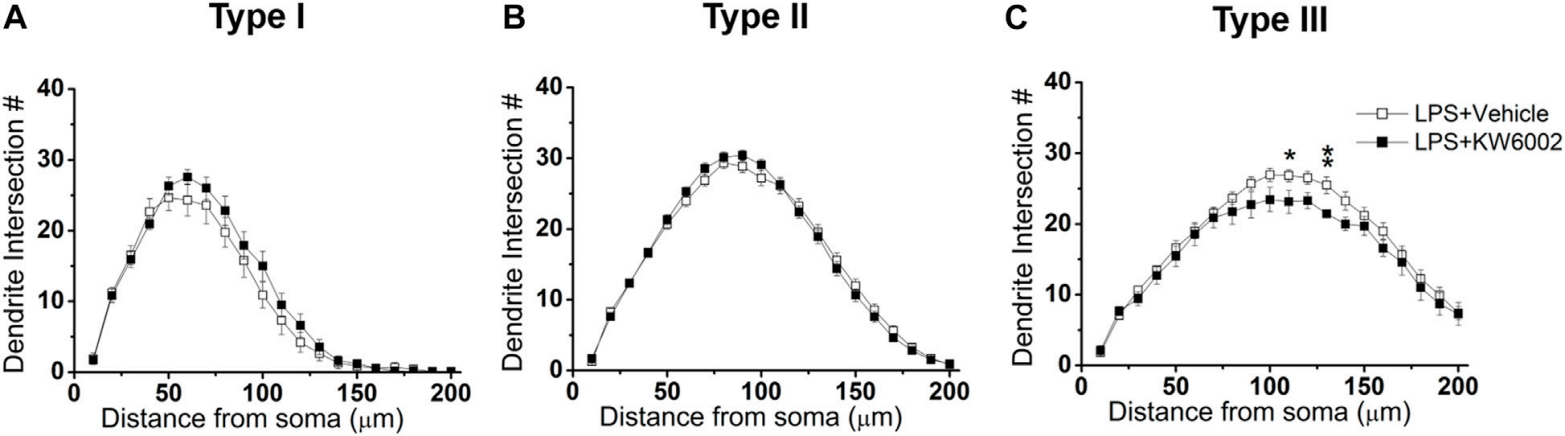
KW6002 decreased the dendritic complexity of Type III RGCs after neonatal LPS exposure. **(A–C)** Quantitative Sholl analysis of dendrite intersection numbers in each RGC type from the vehicle-treated and KW6002-treated group after neonatal LPS exposure. Data represent mean ± SEM. *p < 0.05; **p < 0.01.

We further performed Sholl analyses to study the effect of KW6002 on the spatial distribution of dendritic morphology after neonatal LPS exposure. We found that KW6002 only significantly decline dendritic intersections at 110 and 130 μm from the soma in Type III RGCs (*p < 0.05 or **p < 0.01; Figure 8C), while no significant difference was found either in Type I or Type II RGCs (*p* > 0.05; Figures 8A,B). These results suggest that A_2A_Rs only had slight effect on dendritic complexity after neonatal LPS exposure.

## 4 Discussion

**FIGURE 9:**
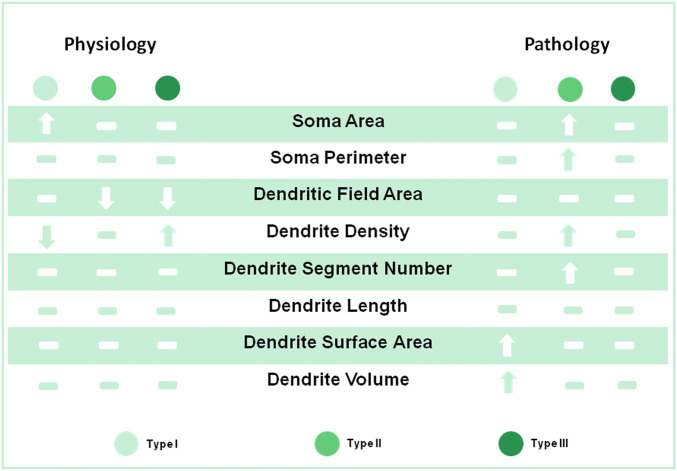
Summary of RGC morphological changes by KW6002 under physiological and pathological conditions The A_2A_R antagonist KW6002 differentially altered these morphological parameters of different RGC types during normal and neonatal inflammation. The arrow means significant up or down-regulation by KW6002, while horizontal line means no significant difference.

The A_2A_R is recently proposed as a potential therapeutic target for retinal diseases (Santiago et al., 2020). However, what exact role of A_2A_R plays in retinal development, especially RGC morphogenesis, is still not be fully elucidated. To simplify the framework for analysis of rather heterogenous RGC types in the retina, here we classified the Thy1-positive RGCs from Thy-1 YFPH transgenic mouse strain into three major morphological types (Type I, II and III) as our previous study (Gao et al., 2018). We found that A_2A_R antagonist KW6002 produced mainly decreased RGC morphogenesis as evident by the reduced dendritic field area of Type II and III, and the reduced dendrite density of Type I but with the increased dendrite density of Type III (Figure 9). The dendritic field represents the input receptive area, while dendrite density represents the intensity of bipolar and amacrine axonal input that RGCs receive within their covered region. Thus A_2A_Rs may modulate the RGC input receptive area and input from bipolar and amacrine in a cell-type specific manner during development. Furthermore, KW6002 had a bidirectional effect on dendritic complexity of Type I and Type III RGCs, suggesting that the fine-tune ability of the A_2A_Rs on the dendritic development of RGCs. Due to lack of an appropriate A_2A_R antibody that reliably and specifically detects A_2A_R in RGCs, whether the different density of A_2A_Rs in the three types of RGCs leads to the distinct effects of KW6002 on the morphology of RGCs remains to be studied in the future. Given the morphology similarity of Type I cells with W3B-RGC (Kim et al., 2010), which is postulated as a selective feature detector (Zhang et al., 2012), Type II cells with ON-sustained alpha RGCs (Bleckert et al., 2014; Krieger et al., 2017; Smeds et al., 2019), and Type III cells with melanopsin M2 cell (Sanes and Masland, 2015), we speculate that A_2A_R activity may potentially modulate local edge detecting (Type I RGC), single-photon visual signal transmission to the brain (Type II RGC) and the function of intrinsically photosensitive melanopsin-containing RGC (Type III RGC). The exact function of three RGCs affected by A_2A_R needs to be characterized by further functional studies.

We further studied the effect of A_2A_R on RGC morphology after neonatal LPS exposure and found that antagonism of A_2A_R changed the compositions of the three RGC types, while no composition change was found in normal development or after neonatal inflammation (Supplementary Figure S1) (Gao et al., 2018). Previous studies have reported that the A_2A_R antagonist prevents RGC loss in retinal organotypic cultures upon exposure to LPS (Madeira et al., 2015) and in several models of retinal neurodegeneration (Madeira et al., 2016; Boia et al., 2017; Aires et al., 2019a; Aires et al., 2019b). It may be possible that A_2A_R antagonists preferrentially protect Type I and II RGCs from death after neonatal inflammation, thus upregulating the proportion of the two types. Notably, after neonatal inflammation, A_2A_R antagonist KW6002 specifically increased the proportion of Type I RGCs with enhanced the dendrite surface area and volume and the proportion of Type II RGCs with enlarged the soma area and perimeter, indicating that the A_2A_R activation exerts mainly suppression on RGC soma and dendrite volume under neonatal inflammation. The increased RGC size of Type I and II by KW6002 may be associated with the upregulation of cell processes such as mitochondrial dynamics to resist cell loss (Miettinen and Bjorklund, 2016). Furthermore, the modulation pattern of A_2A_R antagonist on RGC morphology is quite different from that during normal development (Figure 9), which indicating that A_2A_Rs have distinct effects on RGC morphological development under physiological and pathological conditions. These distinct effects on RGC morphology by KW6002 treatment may attribute to the different local environmental changes. Indeed, different glutamate concentration has been shown to switch the effect of A_2A_R from anti-inflammatory to proinflammatory (Dai et al., 2010). In addition, these distinct effects of A_2A_Rs on RGC morphology may be attributed to different cell types targeted by KW6002. During the normal retinal development, KW6002 may mainly block the A_2A_R on the RGCs, thus affecting the morphological development of RGCs. However, after neonatal inflammation KW6002 may act on the A_2A_R on microglia or both on microglia and RGCs to modulate the morphology of RGCs, since previous studies have found that inflammation can cause a marked increase in microglial A_2A_R (Canas et al., 2004; Wittendorp et al., 2004). Whether the direct effect of KW6002 on microglia or not remains to be determined by future experiment with genetic deleption of the microglial A_2A_R. Our results are in notably agreement with previous studies showing that the complex and differential roles of A_2A_R play under physiological and pathological conditions. For example, we recently found that genetic inactivation of A_2A_R attenuates pathologic angiogenesis in the development of retinopathy of prematurity, but it does not affect developmental angiogenesis in the mouse retina (Liu et al., 2010; Zhang et al., 2017; Zhang et al., 2022). The effect of A_2A_R on the control of peripheral inflammation and chronic neuroinflammation is also opposite (Cunha, 2005). Therefore, A_2A_R signaling may distinctly regulate RGC development under normal and pathological conditions in the retina and the underlying mechanisms need to be further investigated in the future.

Collectively, during development A_2A_R activation can modulate the RGC morphology in a cell type-specific manner and fine-tune the dendritic development by bidirectionally regulating the dendritic complexity of Type I and III RGCs. After neonatal inflammation, A_2A_R activation mainly reduces the soma and dendrites of Type I and II RGCs and diminishes their proportions, which is totally different from the roles it plays during the development. These findings may provide an integrated view of the multi-faced effects of A_2A_R signaling on the morphology of RGCs, which is depending on the cell-types and conditions.

## Data Availability

The original contributions presented in the study are included in the article/Supplementary Material, further inquiries can be directed to the corresponding authors.
