# Supplementary figures and images for: The adenosine A2A receptor antagonist KW6002 distinctly regulates retinal ganglion cell morphology during postnatal development and neonatal inflammation
Source: Front Pharmacol. 2022 Dec 16;13:1082997. doi: 10.3389/fphar.2022.1082997 (PMC9800499; doi:10.3389/fphar.2022.1082997)

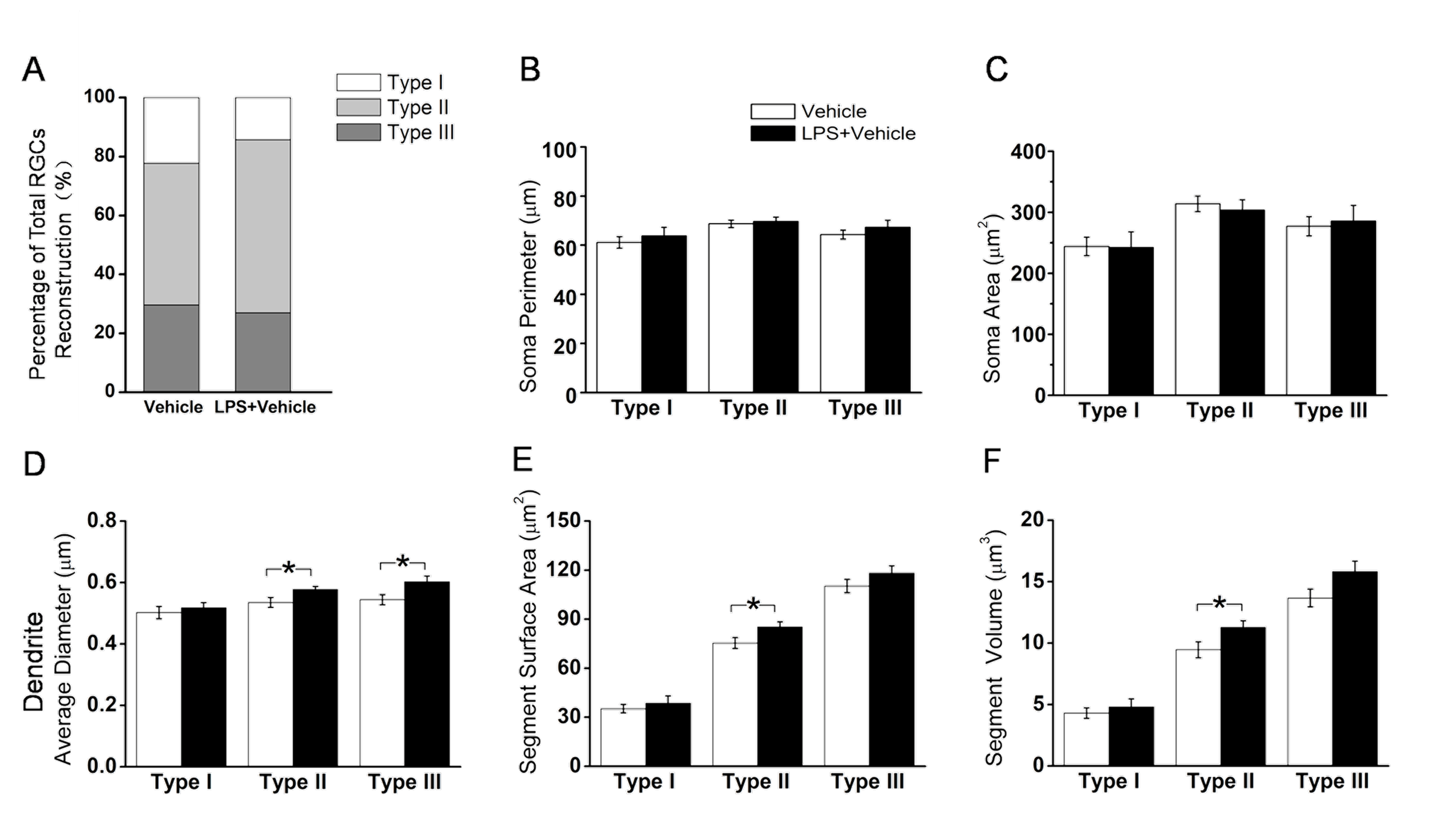

Supplement: Supplementary file 2 [file Image1.TIF]
