# Supplementary material for: The adenosine A2A receptor antagonist KW6002 distinctly regulates retinal ganglion cell morphology during postnatal development and neonatal inflammation
Source: Front Pharmacol. 2022 Dec 16;13:1082997. doi: 10.3389/fphar.2022.1082997 (PMC9800499; doi:10.3389/fphar.2022.1082997)
Supplement: Supplementary file 3 [file DataSheet1.PDF]

**Supplementary Information**

Fig.S1. Neonatal exposure to LPS did not change the proportion of RGC morphological types, but differentially affected the dendritic development of RGCs.

(A) Neonatal exposure to LPS did not alter the composition of the three RGC types.

(B-C) Comparative analysis of soma perimeter (B) and area (C) of different RGC types between the vehicle-treated and neonatal LPS-treated mice.

(D-F) Comparative analysis of average dendrite diameter (D), segment surface area (E) and segment volume (F) of different RGC types between the vehicle-treated and neonatal LPS- treated mice. Data represent mean ± SEM. $*P < 0.05$.
